# Risk of Injurious Fall and Hip Fracture up to 26 y before the Diagnosis of Parkinson Disease: Nested Case–Control Studies in a Nationwide Cohort

**DOI:** 10.1371/journal.pmed.1001954

**Published:** 2016-02-02

**Authors:** Helena Nyström, Anna Nordström, Peter Nordström

**Affiliations:** 1 Geriatric Medicine, Department of Community Medicine and Rehabilitation, Umeå University, Umeå, Sweden; 2 Occupational and Environmental Medicine, Department of Public Health and Clinical Medicine, Umeå University, Umeå, Sweden; Leiden University Medical Center, NETHERLANDS

## Abstract

**Background:**

Low muscle strength has been found in late adolescence in individuals diagnosed with Parkinson disease (PD) 30 y later. This study investigated whether this lower muscle strength also may translate into increased risks of falling and fracture before the diagnosis of PD.

**Methods and Findings:**

Among all Swedish citizens aged ≥50 y in 2005, two nested case–control cohorts were compiled. In cohort I, individuals diagnosed with PD during 1988–2012 (*n* = 24,412) were matched with up to ten controls (*n* = 243,363), and the risk of fall-related injuries before diagnosis of PD was evaluated. In cohort II, individuals with an injurious fall in need of emergency care during 1988–2012 (*n* = 622,333) were matched with one control (*n* = 622,333), and the risk of PD after the injurious fall was evaluated. In cohort I, 18.0% of cases and 11.5% of controls had at least one injurious fall (*p <* 0.001) prior to PD diagnosis in the case. Assessed by conditional logistic regression analysis adjusted for comorbid diagnoses and education level, PD was associated with increased risks of injurious fall up to 10 y before diagnosis (odds ratio [OR] 1.19, 95% CI 1.08–1.31; 7 to <10 y before diagnosis) and hip fracture ≥15 y before diagnosis (OR 1.36, 95% CI 1.10–1.69; 15–26 y before diagnosis). In cohort II, 0.7% of individuals with an injurious fall and 0.5% of controls were diagnosed with PD during follow-up (*p <* 0.001). The risk of PD was increased for up to 10 y after an injurious fall (OR 1.18, 95% CI 1.02–1.37; 7 to <10 y after diagnosis). An important limitation is that the diagnoses were obtained from registers and could not be clinically confirmed for the study.

**Conclusions:**

The increased risks of falling and hip fracture prior to the diagnosis of PD may suggest the presence of clinically relevant neurodegenerative impairment many years before the diagnosis of this disease.

## Abstract 2

In two nested case-control studies using data from a nationwide cohort, Peter Nordström and colleagues assess the risk of injurious fall and hip fracture up to 26 years before the diagnosis of Parkinson's disease.

## Editors' Summary

**Background:**

Parkinson disease (PD) is a common, progressive neurological disease. The disease is caused by the gradual loss of the nerve cells that usually produce dopamine, a neurotransmitter that regulates the body’s movements (motor functions). The symptoms of PD, which develop slowly, include tremor (involuntary trembling of the hands, legs, arm, jaw, and face), slow movement, impaired balance and coordination, and rigidity (muscle stiffness). As these symptoms worsen, affected individuals may have trouble walking, speaking, and swallowing, and they may fall frequently, fracturing bones. No drugs are available to halt the loss of dopamine-producing nerve cells, but medications that replace or mimic the lost dopamine can reduce the severity of these motor symptoms. PD does not directly kill people, and many people with PD have a normal or near-normal life expectancy. However, PD puts a great strain on the body, which makes affected individuals vulnerable to life-threatening infection such as pneumonia.

**Why Was This Study Done?:**

The motor symptoms of PD do not usually develop until people are around 60 years old. However, nonspecific “prodromal” signs (for example, changes in smell perception) can occur many years earlier. Indeed, a recent study reported low muscle strength in late adolescence in patients diagnosed with PD decades later. Could reduced muscular strength be a marker of neuromuscular dysfunction (problems with the nerves that control movement), and could this dysfunction translate into an increased risk of falls and fractures years before PD diagnosis? Here, researchers investigate these questions by undertaking a nested case—control study in a nationwide cohort. A nested case—control study identifies all the individuals in a group (here, everyone living in Sweden who was 50 years old or older at the end of 2005) who have a specific condition (here, PD), identifies several matched individuals (people who are similar to the cases in terms of age, gender, and certain other characteristics) in the same cohort who do not have the condition, and asks whether the cases and controls differ in terms of a specific characteristic (here, falls and fall-related injuries up to 26 years before the diagnosis of PD).

**What Did the Researchers Do and Find?:**

The researchers identified 24,412 Swedish citizens who were diagnosed with PD between 1988 and 2012 by examining the Swedish National Patient Register and matched each case with up to ten controls to form a nested case—control cohort. During an average study period of 20 years prior to a PD diagnosis, 18.0% of cases and 11.5% of controls had at least one fall that caused an injury requiring emergency care. After adjusting for other factors that might affect an individual’s risk of a fall, PD was associated with an increased risk of injurious falls up to ten years before diagnosis (an odds ratio [OR] of 1.19; an OR represents the chance that an outcome will occur given a particular exposure compared to the chance of the outcome occurring in the absence of that exposure) and with an increased risk of hip fracture more than 15 years before diagnosis (OR 1.36; hip fractures are particularly common among people with PD). The strength of these associations was increased closer to PD diagnosis. Finally, in a second nested case—control cohort in which individuals with a record of injurious falls were matched with individuals with no history of falls, the risk of PD was higher among individuals with an injurious fall than among those without during the first ten years after the first recorded fall.

**What Do These Findings Mean?:**

The use of diagnoses obtained from registers that could not be clinically confirmed may limit the accuracy of these findings. Moreover, these findings cannot prove that there is a shared causal link between injurious falls and PD (although the time-dependent changes in the strength of the associations and the results from the second nested case—control cohort strongly suggest a direct link between injurious falls and subsequent PD). However, the finding that the risk of injurious falls—particularly the risk of falls resulting in hip fracture—is increased decades prior to the diagnosis of PD suggests that clinically relevant but subtle neurodegenerative impairment could be present many years before the clinical onset of PD. These findings, which identify potential markers of PD more than a decade before diagnosis, need to be confirmed in other settings but provide new insights into the earliest, hitherto unrecognized, stages of PD.

**Additional Information:**

This list of resources contains links that can be accessed when viewing the PDF on a device or via the online version of the article at http://dx.doi.org/10.1371/journal.pmed.1001954.

- The US National Institute of Neurological Disorders and Stroke provides detailed information about Parkinson disease (in English and Spanish), including links to US organizations that help people with Parkinson disease
- The UK National Health Service Choices website provide information on all aspects of Parkinson disease (including personal stories)
- The UK not-for-profit organization Parkinson’s UK and the US not-for-profit organization National Parkinson Foundation also provide detailed information about Parkinson disease and personal stories
- The UK not-for-profit organization Healthtalk.org provides stories about all aspects of living with Parkinson disease
- MedlinePlus has links to further resources about Parkinson disease (in English and Spanish)
- Wikipedia has a page on nested case—control studies (note that Wikipedia is a free online encyclopedia that anyone can edit; available in several languages)

## Introduction

Fall-related injuries are a major cause of morbidity and mortality in the elderly population [1]. The risk of falling is increased in patients with Parkinson disease (PD) [2,3], and the incidence of hip fracture is particularly high [4], possibly due to dysfunctional balance reactions and impaired ability to protect the hip when falling [5].

PD has an insidious onset; the cardinal motor symptoms are preceded by substantial neurodegeneration [6,7], the timing of which remains uncertain [8,9]. Several studies have reported increased occurrence of nonspecific symptoms years or decades before the diagnosis of PD [8,9]. These prodromal signs reported typically do not involve motor function. Balance impairment has been considered to be a late-stage symptom in PD; it defines the transition to stage 3 on the Hoehn and Yahr scale [10]. However, this staging relies on the clinical evaluation of balance. Quantitative measurements of postural stability have detected abnormalities also in the early stages of PD [11,12], and recent findings suggest that balance can be impaired in the prodromal phase of the disease [13–15]. Whether such subclinical deficits increase the risk of falling remains unclear, but a high incidence of accidental injuries in the years preceding PD diagnosis has been reported [13]. In a recent investigation of the Swedish male population, we found lower muscle strength 30 y before the diagnosis of PD [16]. The effect size of the relation between muscle strength and PD was small, but an association between similarly subtle strength deficits and an increased risk of low-energy fractures has previously been reported [17]. We hypothesized that the reduced muscle strength found in our previous study might be a marker of neuromuscular dysfunction that could translate into an increased risk of falling and fractures a long time before PD diagnosis. Thus, the aim of the present study was to evaluate the risk of injurious falls prior to the clinical onset of PD from a long-term perspective. In a nationwide cohort consisting of all individuals aged ≥50 y, we identified those diagnosed with PD between 1988 and 2012. Using a nested case—control (NCC) design with attention to temporal perspectives, we investigated whether PD diagnosis was preceded by injurious falls in general, and by hip fracture in particular.

## Methods

### Study Population

Permission for the present study was granted by the local ethics committee of Umeå University. The assignment of a unique personal identification number to every Swedish citizen ensures that nationwide registers and databases are highly reliable. The National Patient Register (NPR) provides records of all public inpatient healthcare since 1987, and outpatient consultations in specialist clinics since 2001 [18]. Diagnoses are coded according to the Swedish versions of the International Classification of Diseases (ICD-9 in 1987–1997, ICD-10 since 1998), and the records have shown a high degree of validity, with positive predictive values of 85%–97% [19]. All residents of Sweden aged ≥50 y on December 31, 2005 (*n* = 3,329,400), were considered for inclusion in the present study. Personal identification numbers were used to link data from the NPR with those from other nationwide registers before encoding and depersonalization.

### Diagnoses and Matched Cohort Definition

Within the cohort, we traced diagnoses of PD (ICD-9 code 332A, ICD-10 code G20.9) recorded in the NPR between January 1, 1987, and December 31, 2012. For the same period, we traced medical consultations and hospitalizations due to falls (ICD-9 code E885, ICD-10 codes W00 and W01). In these fall-related care events, we also captured diagnoses of the most common fractures (hip [ICD-9 code 820, ICD-10 codes S72.0–S72.2], wrist [ICD-9 code 813, ICD-10 code S52], lower leg [ICD-9 code 824, ICD-10 code S82], and humerus [ICD-9 code 812, ICD-10 codes S42.2–S42.4]) and head injuries (ICD-9 codes 800–804 and 850–853; ICD-10 codes I62, S02, and S06). Mortality data were obtained from the National Cause of Death Register.

**Fig 1:**
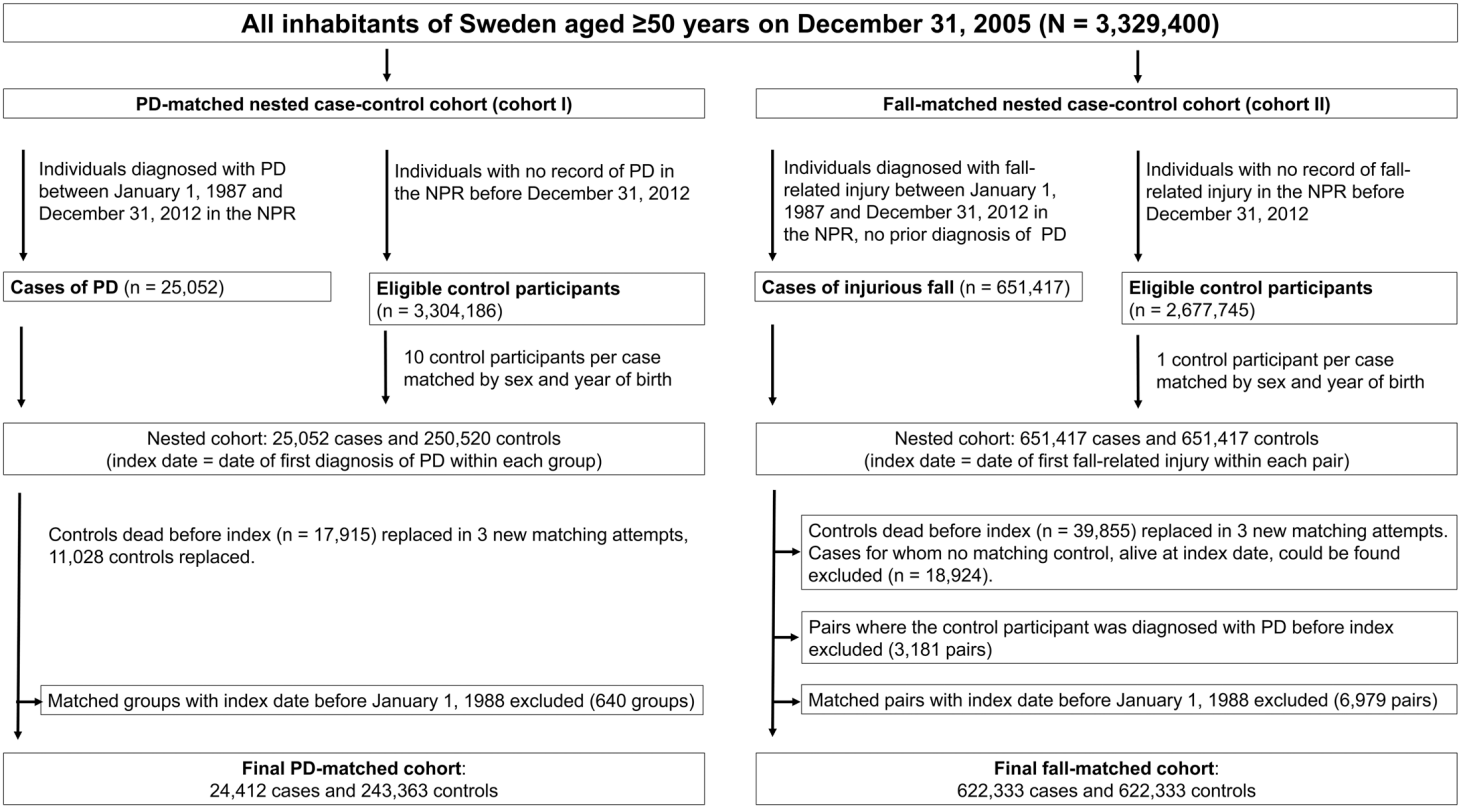
Selection process of the nested case—control cohorts.

Based on the information obtained, we compiled two NCC cohorts (Fig 1). Cohort I was compiled with the aim of analyzing the risk of falls and fractures prior to PD diagnosis compared with matched controls, with a retrospective design allowing capture of recurrent events. For this, each case of PD was matched by sex and year of birth to ten population-based controls, drawn from all residents of Sweden aged ≥50 y on December 31, 2005. The index date for each matched group was defined by the earliest recorded PD diagnosis. Controls deceased before the index date were excluded and were replaced when a new matching control who was alive on the index date could be found within three additional matching attempts. All individuals with index dates before January 1, 1988, were excluded from analyses, to provide a minimum retrospective study time of 1 y and to allow 1 y to capture diagnoses of PD in individuals with disease onset before data collection began.

The second NCC cohort (cohort II) was compiled with the aim of prospectively evaluating the risk of PD after an injurious fall. Again, all residents of Sweden ≥50 y of age by the end of 2005 were considered for inclusion; each individual with a record of injurious fall not preceded by a PD diagnosis in the NPR was matched by sex and year of birth to one control with no record of fall-related injury, drawn from the same population. The index date for each matched pair was defined by the first recorded injurious fall, and pairs containing a control diagnosed with PD before the index date were excluded. Controls deceased before the index date were replaced when possible. Individuals with an injurious fall for whom no matching control alive on the index date could be found within three additional matching attempts were excluded. To allow for 1 y to capture diagnoses of PD, individuals with an index date for injurious fall before January 1, 1988, were excluded from analysis.

### Additional Covariates

Other diagnoses that might confound the associations studied were selected as covariates based on previous known associations with falls [20,21] and were acquired from the NPR, including dementia (ICD-9 codes 290, 291, and 294B; ICD-10 codes F00, F01, F03.9, G30, G31, and E51.2), stroke (ICD-9 codes 431 and 434, ICD-10 codes I61–I64), myocardial infarction (ICD-9 code 410, ICD-10 codes I21 and I22), diabetes mellitus (ICD-9 code 250, ICD-10 codes E10 and E11), depression (ICD-9 code 311, ICD-10 codes F32 and F33), alcohol dependency or abuse (ICD-9 codes 303 and 305A, ICD-10 code F10), and drug dependency or abuse (ICD-9 codes 304 and 305X, ICD-10 codes F11–F19). As a proxy for socioeconomic situation, information about individuals’ education level (low, vocational school or ≤9 y of primary school; high, university or ≥3 y of secondary school) was obtained from the Statistics Sweden database; this information was available for 98.8% of individuals in cohort I and 97.8% of individuals in cohort II.

### Statistical Analyses

Stata (version 13.1 for Windows; StataCorp) and SPSS (version 22.0 for Windows; SPSS Inc.) software packages were used for statistical analyses. Data are presented as valid percentages and medians with ranges, unless otherwise indicated. Chi-squared tests and Wilcoxon rank-sum tests were used for univariate analyses. *p*-Values less than 0.05 were considered significant.

**Fig 2:**
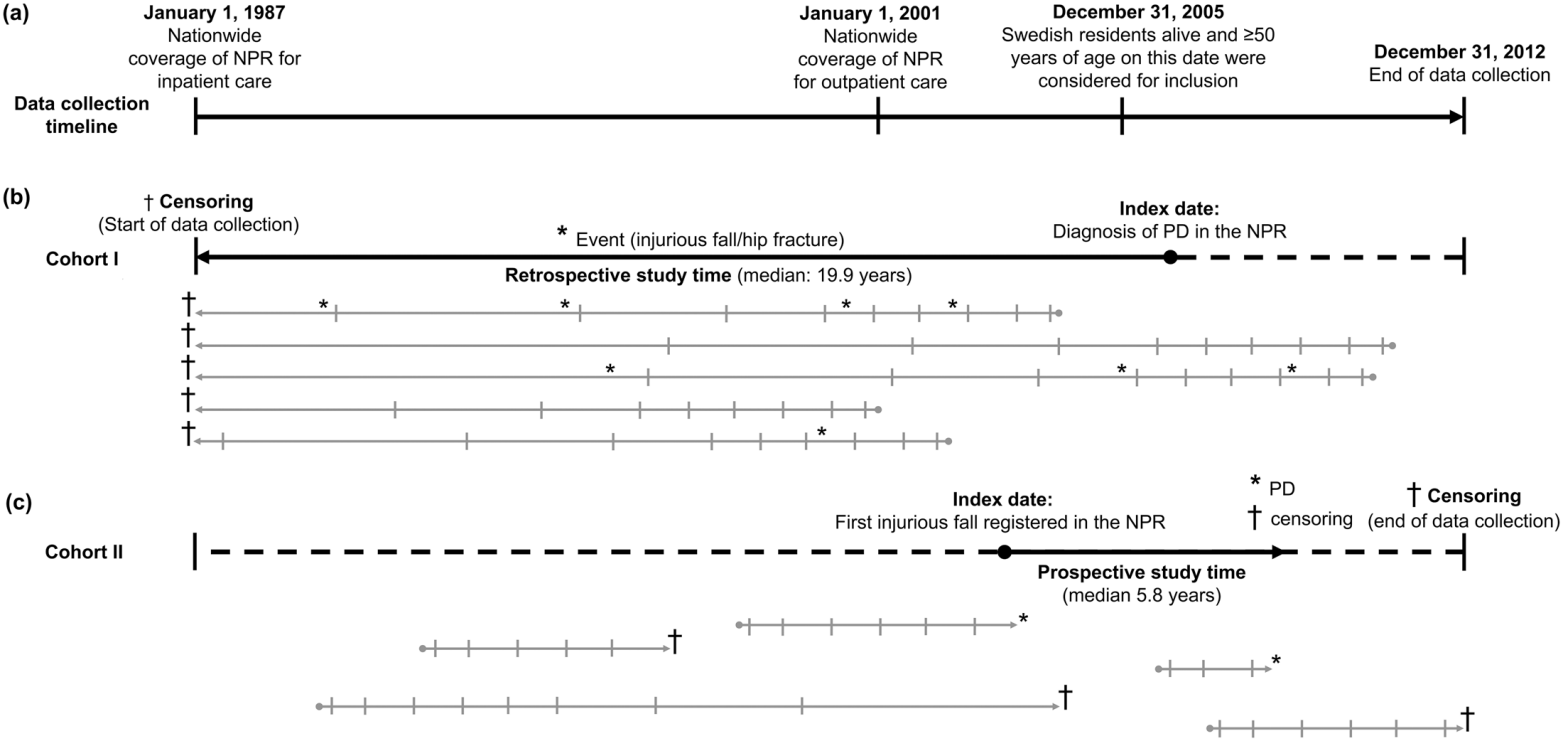
Overview of data collection and study times. (A) The timeline of data collection according to calendar dates. (B) Retrospective analyses in cohort I. The gray arrows provide a schematic illustration of single individuals’ study time, with events (fall/hip fracture) marked (*) above the line. Left censoring (†) occurs at the beginning of the data collection time. Time intervals used in analyses are indicated by horizontal lines (0 to <3 mo, 3 to <12 mo, 1 to <2 y, 2 to <3 y, 3 to <4 y, 4 to <5 y, 5 to <7 y, 7 to <10 y, 10 to <15 y, and 15 to 26 y before the index date). (C) Prospective analyses in cohort II. Individual study times are indicated the same way as in (B), with the follow-up time split into the following intervals after the index date: 0 to <3 mo, 3 to <12 mo, 1 to <2 y, 2 to <3 y, 3 to <4 y, 4 to <5 y, 5 to <7 y, 7 to <10 y, 10 to <15 y, and 15 to 25 y. Marks above the arrow indicate PD (*) or censoring (†) due to death or end of data collection.

In the PD-matched cohort (cohort I), the study time was calculated backward from the index date to January 1, 1987, and incidences of injurious fall and hip fracture were investigated in separate time intervals before the index date: 0 to <3 mo, 3 to <12 mo, 1 to <2 y, 2 to <3 y, 3 to <4 y, 4 to <5 y, 5 to <7 y, 7 to <10 y, 10 to <15 y, and 15 to <26 y before the index date (Fig 2). Medical consultations with aftercare diagnoses (ICD-9 codes V53, V54, and V58; ICD-10 codes Z09.4, Z47, Z48, Z50.8, and Z50.9) were excluded. Odds ratios (ORs) according to PD were calculated separately for each time interval and for each of the outcomes (injurious fall and hip fracture), using conditional logistic regression models adjusted for education level and comorbid diagnoses (and sex and year of birth by the statistical model).

In the fall-matched cohort (cohort II), the follow-up time was calculated from the index date to the date of PD, death, or December 31, 2012, whichever came first. We evaluated the proportional hazards assumption using a Cox model with Schoenfeld residuals and found that the association between injurious fall and subsequent PD did not meet the criteria for proportional hazards (χ^2^ = 156.2 at one degree of freedom; *p <* 0.001). Thus, we further analyzed this association using a flexible parametric Royston—Parmar model [22], allowing the relationship between the exposure and the outcome to vary over time. Individuals with a follow-up time of <3 mo were excluded. To test the consistency of the results, we assessed the ORs for PD in different time intervals using a conditional logistic regression model, as described above. The time intervals for this prospective analysis were 0 to <3 mo, 3 to <12 mo, 1 to <2 y, 2 to <3 y, 3 to <4 y, 4 to <5 y, 5 to <7 y, 7 to <10 y, 10 to <15 y, and 15 to 25 y after the index date. To assess potential confounding by the competing risk of death, we also performed the same analyses separately in a sub-cohort (cohort IIb) where all matched pairs in which either individual died during follow-up were excluded. All models were adjusted for sex and year of birth, education level, and comorbidities diagnosed before the index date.

## Results

Descriptive data for the two NCC cohorts are presented in Table 1. Cohort I comprised 24,412 individuals with PD and 243,363 controls (47.1% women), with a median age of 75.4 y (range 32.0‒103.7) on the index date. During a median study period of 19.9 y (range 1.0–25.0) before the index date, 18.0% of individuals with PD and 11.5% of controls had at least one injurious fall (*p <* 0.001), resulting in at least one fracture in 10.7% of individuals with PD and 6.9% of controls (*p <* 0.001). The incidence of hip fracture prior to the index date was 7.1% and 3.2%, respectively, among individuals with PD and controls (*p <* 0.001; Table 1).

**Table 1:** Descriptive data of the case—control cohorts.

Characteristic	Cohort I, Matched on PD			Cohort II, Matched on Injurious Falls		
	Cases	Controls	*p*-Value	Cases	Controls	*p*-Value
***N***	24,412	243,363		622,333	622,333	
**Study time, retrospective (years)**	19.9 (1.0–26.0)	19.9 (1.0–26.0)	0.525			
**Study time, prospective (years)**				5.5 (0–25.0)	5.9 (0–25.0)	<0.001
**Age at index (years)**	75.5 (32.3‒103.3)	75.4 (32.1‒103.7)	0.383	70.4 (32.1‒106.5)	70.4 (32.1‒106.8)	0.719
**Women**	11,434 (46.8%)	113,931 (46.8%)	0.947	406,688 (65.4%)	406,688 (65.4%)	1.000
**Education level**			<0.001			<0.001
High	6,676 (27.8%)	57,023 (23.7%)		162,418 (26.1%)	164,071 (26.4%)	
Low	17,317 (70.9%)	183,426 (75.4%)		445,690 (71.6%)	444,455 (71.4%)	
Missing	419 (1.7%)	2,914 (1.2%)		14,225 (2.3%)	13,807 (2.2%)	
**Comorbitity** ^a^						
Myocardial infarction	1,585 (6.5%)	18,610 (7.7%)	<0.001	37,203 (6.0%)	32,476 (5.2%)	<0.001
Stroke	1,499 (6.1%)	15,917 (6.5%)	0.016	44,105 (7.1%)	28,169 (4.5%)	<0.001
Diabetes	2,155 (8.8%)	18,597 (7.6%)	<0.001	50,717 (8.2%)	35,292 (5.7%)	<0.001
Dementia	1,461 (6.0%)	6,035 (2.5%)	<0.001	23,265 (3.7%)	10,170 (1.6%)	<0.001
Depression	1,506 (6.2%)	5,105 (2.1%)	<0.001	22,451 (3.6%)	12,371 (2.0%)	<0.001
Alcohol dependency or abuse	382 (1.6%)	3,341 (1.4%)	0.015	23,007 (3.7%)	6,082 (1.0%)	<0.001
Drug dependency or abuse	99 (0.4%)	547 (0.2%)	<0.001	4,163 (0.7%)	1,320 (0.2%)	<0.001
**Incident PD during follow-up**				4,299 (0.7%)	3,202 (0.5%)	<0.001
**Any fall before index date**	4,388 (18.0%)	28,018 (11.5%)	<0.001			
**Fracture** ^b^						
Any fracture	2,612 (10.7%)	16,727 (6.9%)	<0.001	303,376 (48.8%)		
Hip fracture	1,729 (7.1%)	7,757 (3.2%)	<0.001	98,612 (15.9%)		
Wrist fracture	595 (2.4%)	4,829 (2.0%)	<0.001	93,056 (15.0%)		
Lower leg fracture	336 (1.4%)	3,855 (1.6%)	0.013	74,027 (11.9%)		
Humerus fracture	424 (1.7%)	2,725 (1.1%)	<0.001	43,653 (7.0%)		
**Head injury** ^b^	428 (1.8%)	2,799 (1.2%)	<0.001	44,797 (7.2%)		

Data are given as median (range) or number (percent).

^a^In cohort I: at any time during the study; in cohort II: only diagnoses recorded before the index date are included.

^b^In cohort I: at any time during the study; in cohort II: at first fall.

In the multivariable adjusted analyses of cohort I (Table 2), an elevated risk of falls appeared in the decade before PD diagnosis (OR 1.19, 95% CI 1.08–1.31; 7 to <10 y before diagnosis, for cases versus controls), and the association was further accentuated closer to the index date (OR 1.94, 95% CI 1.79–2.11; 3 to <12 mo before diagnosis). The association between PD and hip fracture was even stronger, with an approximately 2-fold increased risk in all intervals between 3 mo and <4 y prior to PD diagnosis, and this association was significant ≥15 y before diagnosis (OR 1.36, 95% CI 1.10–1.69; 15 to 26 y before PD diagnosis). In the last 3 mo before PD diagnosis, the ORs for injurious fall and for hip fracture increased distinctly, to 5.83 (95% CI 5.30–6.42) for falls and 7.49 (95% CI 6.52–8.61) for hip fracture, for individuals with PD versus controls.

**Table 2:** Incidence and odds ratios of injurious fall and hip fracture in cohort I.

Event	Time Interval	Individuals at Risk at Beginning of Interval		Events within Interval		*p*-Value	Adjusted OR (95% CI) according to PD	*p*-Value
		PD	Control	PD	Control			
**Any injurious fall**								
	≥15 y prior to index	18,175	180,993	266 (1.5%)	2,851 (1.6%)	0.248	0.91 (0.80–1.04)	0.174
	10 to <15 y prior to index	19,999	199,233	347 (1.7%)	3,562 (1.8%)	0.591	0.94 (0.84–1.06)	0.304
	7 to <10 y prior to index	21,691	216,153	506 (2.3%)	4,205 (2.0%)	<0.001	1.19 (1.08–1.31)	<0.001
	5 to <7 y prior to index	22,752	226,763	564 (2.5%)	4,339 (1.9%)	<0.001	1.25 (1.14–1.37)	<0.001
	4 to <5 y prior to index	23,217	231,413	409 (1.8%)	2,791 (1.2%)	<0.001	1.40 (1.26–1.56)	<0.001
	3 to <4 y prior to index	23,594	235,183	533 (2.3%)	3,297 (1.4%)	<0.001	1.54 (1.40–1.70)	<0.001
	2 to <3 y prior to index	23,979	239,033	645 (2.7%)	3,917 (1.6%)	<0.001	1.61 (1.47–1.76)	<0.001
	1 to <2 y prior to index	24,409	243,333	773 (3.2%)	4,463 (1.8%)	<0.001	1.69 (1.56–1.83)	<0.001
	3 to <12 mo prior to index	24,412	243,363	763 (3.1%)	3,873 (1.6%)	<0.001	1.94 (1.79–2.11)	<0.001
	<3 mo prior to index	24,412	243,363	816 (3.3%)	1,503 (0.6%)	<0.001	5.83 (5.30–6.42)	<0.001
**Hip fracture**								
	≥15 y prior to index	18,175	180,993	100 (0.6%)	732 (0.4%)	0.004	1.36 (1.10–1.69)	0.004
	10 to <15 y prior to index	19,999	199,233	161 (0.8%)	1,159 (0.6%)	<0.001	1.37 (1.15–1.63)	<0.001
	7 to <10 y prior to index	21,691	216,153	226 (1.0%)	1,273 (0.6%)	<0.001	1.83 (1.58–2.12)	<0.001
	5 to <7 y prior to index	22,752	226,763	231 (1.0%)	1,328 (0.6%)	<0.001	1.72 (1.49–1.99)	<0.001
	4 to <5 y prior to index	23,217	231,413	167 (0.7%)	930 (0.4%)	<0.001	1.78 (1.50–2.11)	<0.001
	3 to <4 y prior to index	23,594	235,183	219 (0.9%)	1,051 (0.5%)	<0.001	2.01 (1.73–2.35)	<0.001
	2 to <3 y prior to index	23,979	239,033	278 (1.2%)	1,271 (0.5%)	<0.001	2.20 (1.92–2.52)	<0.001
	1 to <2 y prior to index	24,409	243,333	313 (1.3%)	1,476 (0.6%)	<0.001	2.11 (1.85–2.40)	<0.001
	3 to <12 mo prior to index	24,412	243,363	287 (1.2%)	1,339 (0.6%)	<0.001	2.15 (1.88–2.45)	<0.001
	<3 mo prior to index	24,412	243,363	393 (1.6%)	529 (0.2%)	<0.001	7.49 (6.52–8.61)	<0.001

ORs for injurious falls and for hip fractures in cohort I for individuals with PD compared to controls, investigated by conditional logistic regression model adjusted for education level and comorbid diagnoses (dementia, stroke, myocardial infarction, diabetes mellitus, depression, alcohol dependency or abuse, drug dependency or abuse).

**Fig 3:**
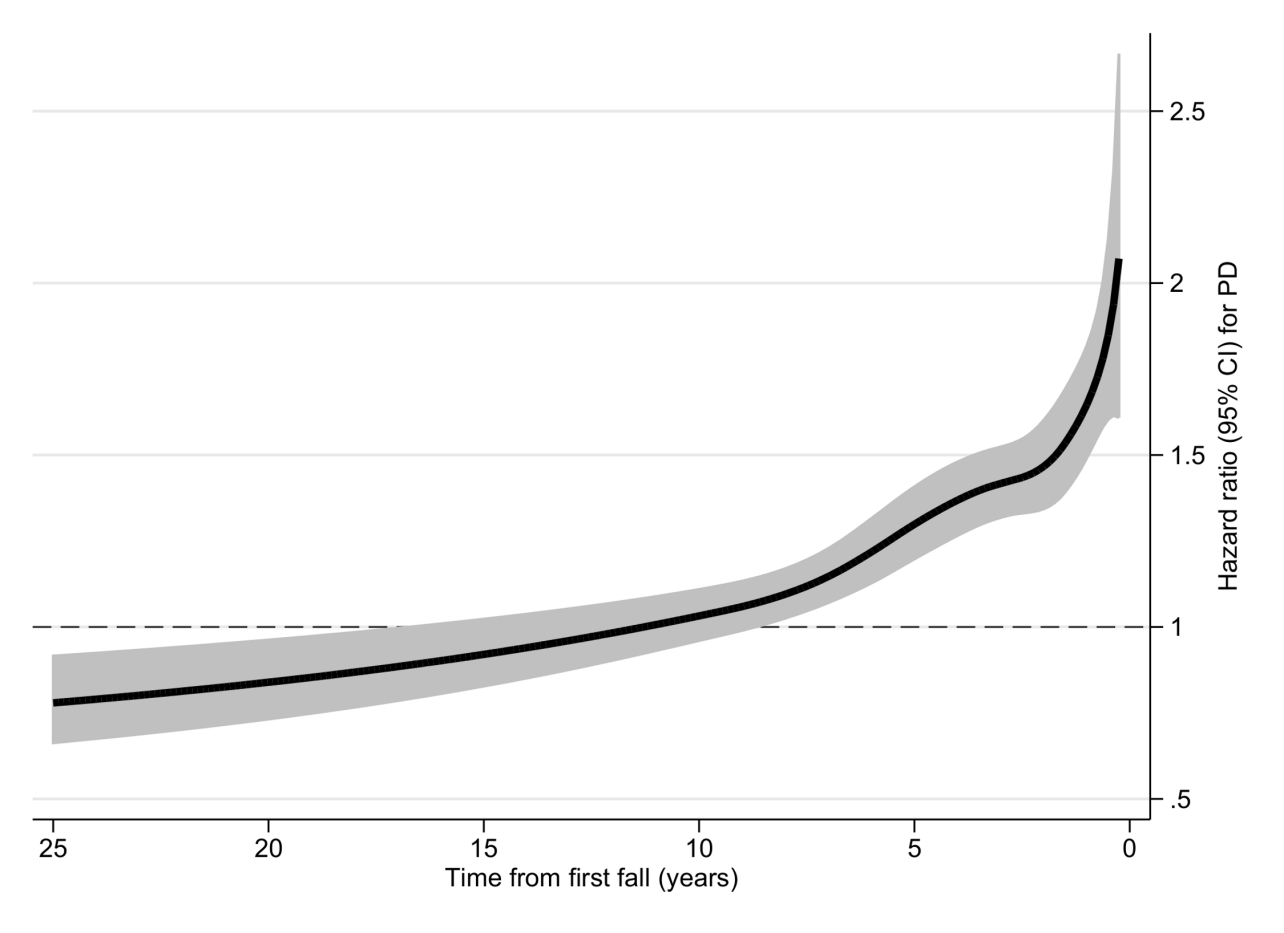
Hazard ratio for Parkinson disease according to fall in cohort II. Hazard ratio for PD after first injurious fall in cohort II, estimated by a flexible parametric Royston—Parmar model adjusted for sex, year of birth, education level, and comorbid diagnoses (dementia, stroke, myocardial infarction, diabetes mellitus, depression, alcohol dependency or abuse, drug dependency or abuse). The gray areas represent the 95% confidence intervals.

Results of the prospective sensitivity analyses of risk of PD after injurious fall in cohort II are presented in Fig 3 (Royston—Parmar model) and Table 3 (conditional logistic regression). Cohort II consisted of 622,333 individuals with an injurious fall and 622,333 individuals without an injurious fall (65.4% women) with a median age of 70.4 y (range 32.1‒106.8) at the time of the first registered injurious fall. The most common fall-related injuries were fractures of the hip (15.9%), wrist (15.0%), lower leg (11.9%), and humerus (7.0%). During the follow-up period (median 5.8 y, range 0–25.0), 7,501 (0.6%) individuals in this cohort were diagnosed with PD (4,299 [0.7%] individuals with an injurious fall, 3,202 [0.5%] controls; *p <* 0.001; Table 1).

**Table 3:** Incidence of Parkinson disease according to fall in cohort II.

Time Interval	Individuals at Risk at Beginning of Interval		PD within Interval		*p*-Value	Adjusted OR for PD (95% CI), according to Fall	*p*-Value
	Injurious Fall	No Injurious Fall	Injurious Fall	No Injurious Fall			
≥15 y after index	70,249	73,376	218 (0.31%)	277 (0.38%)	0.030	0.79 (0.65–0.95)	0.015
10 to <15 y after index	151,308	158,874	329 (0.22%)	386 (0.24%)	0.149	0.88 (0.75–1.03)	0.117
7 to <10 y after index	251,614	264,762	443 (0.18%)	420 (0.16%)	0.125	1.18 (1.02–1.37)	0.028
5 to <7 y after index	333,831	351,582	487 (0.15%)	408 (0.12%)	0.001	1.34 (1.15–1.55)	<0.001
4 to <5 y after index	380,662	399,912	309 (0.08%)	242 (0.06%)	0.001	1.45 (1.19–1.75)	<0.001
3 to <4 y after index	431,866	451,736	397 (0.09%)	307 (0.07%)	<0.001	1.41 (1.20–1.67)	<0.001
2 to <3 y after index	487,169	506,491	454 (0.09%)	353 (0.07%)	<0.001	1.36 (1.17–1.59)	<0.001
1 to <2 y after index	547,228	564,848	565 (0.10%)	389 (0.07%)	<0.001	1.50 (1.30–1.73)	<0.001
3 to <12 mo after index	595,267	607,462	533 (0.09%)	291 (0.05%)	<0.001	1.98 (1.70–2.32)	<0.001
<3 mo after index	622,333	622,333	564 (0.09%)	129 (0.02%)	<0.001	4.31 (3.52–5.29)	<0.001

ORs for PD after injurious fall in cohort II, investigated by conditional logistic regression model adjusted for education level and comorbid diagnoses (dementia, stroke, myocardial infarction, diabetes mellitus, depression, alcohol dependency or abuse, drug dependency or abuse).

Assessed by the Royston—Parmar model, the risk of PD was elevated among individuals with an injurious fall during the first ~10 y of study enrollment and then appeared to decrease significantly compared with controls at follow-up times ≥15 y (Fig 3). Regression analyses confirmed these findings, with ORs declining from 1.98 (95% CI 1.70–2.32) at 3 to <12 mo after the first fall to 0.79 (95% CI 0.65–0.95) at ≥15 y after the first fall (Table 3). However, in the sub-cohort excluding all pairs censored by death (S1 Table), no inverse relationship between falling and PD was observed (S2 Table; S1 Fig).

## Discussion

To our knowledge, this study is the first long-term investigation of fall risk prior to the clinical onset of PD. In general, we found that the risk of injurious falls was elevated a decade before the diagnosis of PD, and the risk of hip fracture was increased ≥15 y before the diagnosis of PD. The strength of these associations between injurious falls, hip fracture, and PD increased distinctly up to the diagnosis of PD. The time-dependent patterns indicate a direct link between injurious falls and subsequent PD, and may be explained by subtle neurodegenerative impairment many years before the diagnosis of this disease.

In the present study, diagnoses of dementia, depression, and diabetes were more common in PD cases than in controls, which may have contributed to the higher risk of falls before the index date [20,21]. Irrespectively of this, a recent study from the UK revealed that balance impairment was more common up to 5 y prior to diagnosis in individuals diagnosed with PD compared to controls [14]. This finding is particularly noteworthy, as postural imbalance is not expected to be clinically overt until the later stages of PD [11,23]. Moreover, given that subclinical deficits in postural stability have been noted in early PD [11,12], similar findings of increased postural sway in patients with rapid eye movement sleep behavior disorder may indicate balance impairments in prodromal PD, as more than half of these patients are expected to eventually develop parkinsonism (PD, multiple system atrophy, or Lewy body dementia) [15]. Such subtle deficits could conceivably translate into increased risks of falling and consequent injury in prodromal PD, but, to our knowledge, only one previous study [13] has addressed this question. The authors reported an increased incidence of accidental injury prior to PD but included data from only the last 3 y of the prodromal phase [13], whereas our results indicate an association with hip fracture decades before diagnosis.

The results of the present study are supported by previous findings of low hand-grip and elbow-flexion strength three decades before the diagnosis of PD [16]. The involvement of the upper extremity is of particular interest in relation to the increased risk of hip fracture in the present study, as dysfunctional arm movements when falling are thought to play an important role in the high incidence of hip fracture in patients with PD [2]. Thus, taken together, our present and previous [16] findings may indicate that such deficits arise long before the cardinal signs of PD become overt.

Some limitations of the present study should be noted. First, data were obtained from registers, and diagnoses could not be clinically confirmed for the present study, although all diagnoses were recorded in the context of specialist healthcare. Compared to residents of urban areas, individuals with PD in rural parts of Sweden may be less likely to be diagnosed in specialist health care, and therefore less likely to be included as cases of PD in the present study. A similar effect could be expected for fall-related injuries; less severe injuries that do not demand surgery are less likely to be diagnosed in specialist care. Thus, the accuracy for hip fractures can be expected to be higher than for fall-related injuries in general [24]. Any incorrect or missed diagnosis would contribute to regression dilution bias, causing wider standard errors and attenuation of the associations found between PD and injurious falls toward zero [25]. Second, the observational nature of this study did not permit exploration of the mechanisms underlying the observed associations or documentation of a causal link between injurious falls and PD, although the time-dependent associations found strongly suggest such a relationship. Third, we lacked information about smoking status. This would have been of interest since smoking is associated with a reduced risk for PD [26]. Having a higher education level is known to be associated with less smoking [27,28], and smoking status may therefore explain the association between higher education and PD in the present study. The main strengths of the present study include the large body of prospectively recorded data covering a long period, which provided superior statistical power for the performance of reliable analyses from a long-term perspective and which was not subject to recall bias. The main results of the present study were also evaluated in cohort II, and the findings were consistent. Thus, the sensitivity analysis supported the validity of the main findings, and because the individuals were drawn from a nationwide cohort, the external validity of the results is likely high.

In conclusion, we found that the risk of injurious falls in general, and especially those resulting in hip fracture, was increased decades before the diagnosis of PD. These findings suggest that clinically relevant neurodegenerative impairment could be present many years before the clinical onset of the disease. It would be of value if our results identifying markers of PD more than a decade before diagnosis, from the present and previous studies [16,29], could be confirmed in other settings and in other countries.

## Supporting Information

S1 Fig Hazard ratio for Parkinson disease according to fall in cohort II excluding all matched pairs censored by death (cohort IIb). Hazard ratio estimated by a flexible parametric Royston—Parmar model adjusted for sex, age at index date, education level, and comorbid diagnoses (dementia, stroke, myocardial infarction, diabetes mellitus, depression, alcohol dependency or abuse, drug dependency or abuse). The gray areas represent the 95% confidence intervals.
(TIFF)

S1 Strobe Checklist (DOC)

S1 Table Descriptive data of cohort II after excluding all matched pairs censored by death during the study time (cohort IIb). (DOCX)

S2 Table Odds ratio for Parkinson disease according to fall after excluding all matched pairs censored by death during the study time in cohort II (cohort IIb). OR estimated by conditional logistic regression model adjusted for education level and comorbid diagnoses (dementia, stroke, myocardial infarction, diabetes mellitus, depression, alcohol dependency or abuse, drug dependency or abuse).
(DOCX)

S1 Text History of analysis plan. (DOCX)

## Abbreviations

NCC: nested case—control
NPR: National Patient Register
OR: odds ratio
PD: Parkinson disease
