# Supplementary figures and images for: Risk of Injurious Fall and Hip Fracture up to 26 y before the Diagnosis of Parkinson Disease: Nested Case–Control Studies in a Nationwide Cohort
Source: PLoS Med. 2016 Feb 2;13(2):e1001954. doi: 10.1371/journal.pmed.1001954 (PMC4737490; doi:10.1371/journal.pmed.1001954)

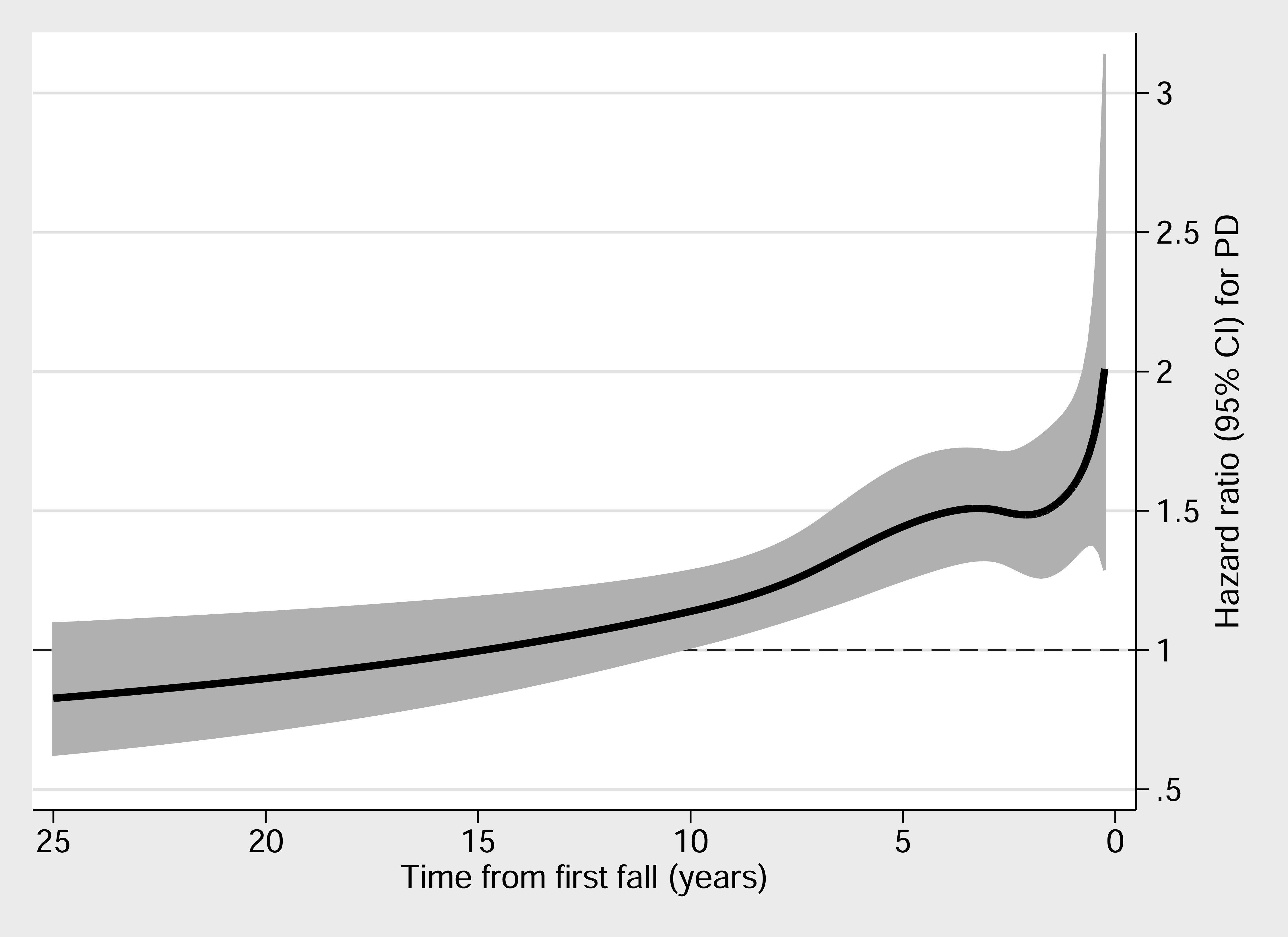

Supplement: S1 Fig — Hazard ratio estimated by a flexible parametric Royston—Parmar model adjusted for sex, age at index date, education level, and comorbid diagnoses (dementia, stroke, myocardial infarction, diabetes mellitus, depression, alcohol dependency or abuse, drug dependency or abuse). The gray areas represent the 95% confidence intervals. (TIFF) [file pmed.1001954.s001.tiff]
