# Supplementary material for: Risk of Injurious Fall and Hip Fracture up to 26 y before the Diagnosis of Parkinson Disease: Nested Case–Control Studies in a Nationwide Cohort
Source: PLoS Med. 2016 Feb 2;13(2):e1001954. doi: 10.1371/journal.pmed.1001954 (PMC4737490; doi:10.1371/journal.pmed.1001954)
Supplement: S1 Strobe Checklist — (DOC) [file pmed.1001954.s002.doc]

**STROBE 2007 (v4) checklist of items to be included in reports of observational studies in epidemiology***

**Checklist for cohort, case-control, and cross-sectional studies (combined)**

| **Section/Topic** | Item # | Recommendation | Reported on page # |
| --- | --- | --- | --- |
| **Title and abstract** | 1 | (*a*) Indicate the study’s design with a commonly used term in the title or the abstract | **Title**: “Risk of injurious fall and hip fracture up to 26 years before the diagnosis of Parkinson’s disease: nested case-control studies in a nationwide cohort” |
|  |  | (*b*) Provide in the abstract an informative and balanced summary of what was done and what was found | **Abstract**: “Among all Swedish citizens aged ≥50 years in 2005, 24,412 individuals were diagnosed with PD during 1988–2012. Each of these persons were matched with up to 10 controls based on sex and year of birth (n=243,363) to form a nested case-control cohort. In this cohort, associations between PD and previous fall-related injuries requiring emergency care were evaluated by multivariable adjusted statistical models with attention to time perspectives.  During a median study period of 20 years prior to PD diagnosis, 18.0% of cases and 11.5% of controls had at least 1 injurious fall (p<0.001). Assessed by conditional logistic regression analysis adjusted for comorbid diagnoses and education level, PD was associated with increased risks of injurious fall up to 10 years before diagnosis [odds ratio (OR) 1.19, 95% confidence interval (CI) 1.08–1.31; 7–10 years before diagnosis] and hip fracture >15 years before diagnosis (OR 1.36, 95% CI 1.10–1.69; 15–26 years before diagnosis). The strength of both associations increased closer to PD diagnosis; at 3–12 months before diagnosis, the OR for injurious fall was 1.94 (95% CI 1.79–2.11) and that for hip fracture was 2.15 (95% CI 1.88–2.45) for cases compared with controls. An important limitation is that the diagnoses were obtained from registers and could not be clinically confirmed for the study.” |
| Introduction | | |  |
| Background/rationale | 2 | Explain the scientific background and rationale for the investigation being reported | **Abstract**: “We hypothesized that the reduced muscle strength found in our previous study might be a marker of neuromuscular dysfunction which could translate into an increased risk of falling and fractures a long time before PD diagnosis.” |
| Objectives | 3 | State specific objectives, including any pre-specified hypotheses | **Introduction, last paragraph:** “Using a nested case-control (NCC) design with attention to temporal perspectives, we investigated whether PD diagnosis was preceded by injurious falls in general, and by hip fracture in particular.” |
| Methods | | |  |
| Study design | 4 | Present key elements of study design early in the paper | **Abstract**: “Among all Swedish citizens aged ≥50 years in 2005, 24,412 individuals were diagnosed with PD during 1988–2012. Each of these persons were matched with up to 10 controls based on sex and year of birth (n=243,363) to form a nested case-control cohort.”  **Methods, 1^st^ paragraph:** “The National Patient Register (NPR) provides records of all public inpatient health care since 1987, and outpatient consultations in specialist clinics since 2001.”  “All residents of Sweden aged ≥50 years on December 31, 2005 (N=3,329,400) were considered for inclusion in the present study.”  **Methods, 2^nd^ paragraph:** “Within the cohort, we traced diagnoses of PD (ICD-9 code 332A, ICD-10 code G20.9) recorded in the NPR between January 1, 1987 and December 31, 2012. For the same period, we traced medical consultations and hospitalizations due to falls in the same level” |
| Setting | 5 | Describe the setting, locations, and relevant dates, including periods of recruitment, exposure, follow-up, and data collection | **Methods, 1^st^ paragraph:** “The National Patient Register (NPR) provides records of all public inpatient health care since 1987, and outpatient consultations in specialist clinics since 2001.”  “All residents of Sweden aged ≥50 years on December 31, 2005 (N=3,329,400) were considered for inclusion in the present study.”  **Methods, 2^nd^ paragraph:** “Within the cohort, we traced diagnoses of PD (ICD-9 code 332A, ICD-10 code G20.9) recorded in the NPR between January 1, 1987 and December 31, 2012. For the same period, we traced medical consultations and hospitalizations due to falls in the same level”  **Methods, 3^rd^ paragraph:** Cohort I: “…each case of PD was matched by sex and year of birth to 10 population based controls, drawn from all residents of Sweden aged ≥50 years on December 31, 2005. The index date for each matched group was defined by the earliest recorded PD diagnosis”, “In the PD-matched cohort (cohort I), the study time was calculated backward from the index date to January 1, 1987, and incidences of injurious fall and hip fracture were investigated in separate time intervals based on the index date”  **Methods, 4^th^ paragraph:** Cohort II: “…each individual with a record of injurious fall, not preceded by a PD diagnosis in the NPR, was matched by sex and year of birth to 1 control with no record of fall-related injury, drawn from the same population. The index date for each matched pair was defined by the first recorded injurious fall”, “In the fall-matched cohort (cohort II), the follow-up time was calculated from the index date to the date of PD, death, or December 31, 2012, whichever came first.” |
| Participants | 6 | (*a*) *Cohort study*—Give the eligibility criteria, and the sources and methods of selection of participants. Describe methods of follow-up  *Case-control study*—Give the eligibility criteria, and the sources and methods of case ascertainment and control selection. Give the rationale for the choice of cases and controls  *Cross-sectional study*—Give the eligibility criteria, and the sources and methods of selection of participants | **Methods**; for text extracts please see point 5 above. |
|  |  | (*b*) *Cohort study*—For matched studies, give matching criteria and number of exposed and unexposed  *Case-control study*—For matched studies, give matching criteria and the number of controls per case | **Methods, 2^nd^ paragraph:** “Within the cohort, we traced diagnoses of PD (ICD-9 code 332A, ICD-10 code G20.9) recorded in the NPR between January 1, 1987 and December 31, 2012. For the same period, we traced medical consultations and hospitalizations due to falls in the same level (ICD-9 code E885, ICD-10 codes W00 and W01).”  **Methods, 3^rd^ paragraph:** Cohort I: “…each case of PD was matched by sex and year of birth to 10 population based controls, drawn from all residents of Sweden aged ≥50 years on December 31, 2005.”  **Methods, 4^th^ paragraph:** Cohort II “…each individual with a record of injurious fall, not preceded by a PD diagnosis in the NPR, was matched by sex and year of birth to 1 control with no record of fall-related injury, drawn from the same population.” |
| Variables | 7 | Clearly define all outcomes, exposures, predictors, potential confounders, and effect modifiers. Give diagnostic criteria, if applicable | **Methods, 2^nd^ paragraph:** “diagnoses of PD (ICD-9 code 332A, ICD-10 code G20.9)”; “falls in the same level (ICD-9 code E885, ICD-10 codes W00 and W01)”  **Methods, 5^th^ paragraph:** “most common fractures [hip (ICD-9 code 820, ICD-10 codes S72.0–S72.2), wrist (ICD-9 code 813, ICD-10 code S52), lower leg (ICD-9 code 824, ICD-10 code S82), and humerus (ICD-9 code 812, ICD-10 codes S42.2–S42.4)] and head injuries (ICD-9 codes 800–804 and 850–853; ICD-10 codes I62, S02, and S06).”  **Methods, 7^th^ paragraph:** “Medical consultations with aftercare diagnoses (ICD-9 codes V53, V54, and V58; ICD-10 codes Z09.4, Z47, Z48, Z50.8, and Z50.9) were excluded.”  **Methods, 5^th^ paragraph:** Potential confounders:  “Other diagnoses that might confound the associations studied were selected based on previous known associations with falls[20, 21] and acquired from the NPR, including dementia (ICD-9 codes 290, 291, and 294B; ICD-10 codes F00, F01, F03.9, G30, G31, and E51.2), stroke (ICD-9 codes 431 and 434, ICD-10 codes I61–I64), myocardial infarction (ICD-9 code 410, ICD-10 codes I21 and I22), diabetes mellitus (ICD-9 code 250, ICD-10 codes E10 and E11), depression (ICD-9 code 311, ICD-10 codes F32 and F33), alcohol dependency or abuse (ICD-9 codes 303 and 305A, ICD-10 code F10), and drug dependency or abuse (ICD-9 codes 304 and 305X, ICD-10 codes F11–F19). As a proxy for socioeconomic situation, information about individuals’ education level (low, vocational school or ≤9 years primary school; high, university or ≥3 years secondary school) was obtained from the Statistics Sweden database; this information was available for 98.8% of individuals in cohort I and 97.8% of individuals in cohort II.” |
| Data sources/ measurement | 8* | For each variable of interest, give sources of data and details of methods of assessment (measurement). Describe comparability of assessment methods if there is more than one group | **Methods, 1^st^ paragraph: “**The National Patient Register (NPR) provides records of all public inpatient healthcare since 1987, and outpatient consultations in specialist clinics since 2001”  **Methods, 2^nd^ paragraph:** “Mortality data were obtained from the National Cause of Death Register.”  **Methods, 5^th^ paragraph: “**Other diagnoses that might confound the associations studied were selected based on previous known associations with falls[20, 21] and acquired from the NPR”  “As a proxy for socioeconomic situation, information about individuals’ education level (low, vocational school or ≤9 years primary school; high, university or ≥3 years secondary school) was obtained from the Statistics Sweden database; this information was available for 98.8% of individuals in cohort I and 97.8% of individuals in cohort II.” |
| Bias | 9 | Describe any efforts to address potential sources of bias | **Methods, 5^th^ paragraph:** “Other diagnoses that might confound the associations studied were selected based on previous known associations with falls”  **Methods, 8^th^ paragraph:** “To assess potential confounding by the competing risk of death, we also performed the same analyses separately in a sub cohort (cohort IIb) where all matched pairs in which any individual died during follow up were excluded. All models were adjusted for sex and year of birth, education level, and comorbidities diagnosed before the index date.” |
| Study size | 10 | Explain how the study size was arrived at | **Methods, paragraph 1-3:** “All residents of Sweden aged ≥50 years on December 31, 2005 (N=3,329,400) were considered for inclusion in the present study.”  “each case of PD was matched by sex and year of birth to 10 population based controls,”  “each individual with a record of injurious fall, not preceded by a PD diagnosis in the NPR, was matched by sex and year of birth to one control” |
| Quantitative variables | 11 | Explain how quantitative variables were handled in the analyses. If applicable, describe which groupings were chosen and why | **Methods, 6^th^ paragraph:** “Data are presented as valid percentages and medians with ranges, unless otherwise indicated. Chi-squared tests and Wilcoxon rank-sum tests were used for univariate analyses.” |
| Statistical methods | 12 | (*a*) Describe all statistical methods, including those used to control for confounding | **Methods, 6^th^ paragraph:** “Data are presented as valid percentages and medians with ranges, unless otherwise indicated. Chi-squared tests and Wilcoxon rank-sum tests were used for univariate analyses.”  **Methods, 7^th^ paragraph:** “Odds ratios (ORs), according to PD, were calculated separately for each time interval and for each of the outcomes (injurious fall and hip fracture, respectively), using conditional logistic regression models adjusted for education level and comorbid diagnoses (and sex and year of birth by the statistical model).”  **Methods, 8^th^ paragraph:** “We evaluated the proportional hazards assumption using a Cox model with Schoenfeld residuals, and found that the association between injurious fall and subsequent PD did not meet the criteria for proportional hazards (Chi2 = 156.2 at 1 degree of freedom; p<0.001). Thus, we further analyzed this association using a flexible parametric Royston–Parmar model, allowing the relationship between the exposure and the outcome to vary over time.”  “To test the consistency of the results, we assessed the ORs for PD in different time intervals using a conditional logistic regression model, as described above.” |
|  |  | (*b*) Describe any methods used to examine subgroups and interactions | **Methods, 8^th^ paragraph:** “To assess potential confounding by the competing risk of death, we also performed the same analyses separately in a sub cohort (cohort IIb) where all matched pairs in which any individual died during follow up were excluded. All models were adjusted for sex and year of birth, education level, and comorbidities diagnosed before the index date.” |
|  |  | (*c*) Explain how missing data were addressed | Data were only missing for education, and only for less than 3% of the population.  **Methods, 5^th^ paragraph:** “…this information was available for 98.8% of individuals in cohort I and 97.8% of individuals in cohort II.” |
|  |  | (*d*) *Cohort study*—If applicable, explain how loss to follow-up was addressed  *Case-control study*—If applicable, explain how matching of cases and controls was addressed  *Cross-sectional study*—If applicable, describe analytical methods taking account of sampling strategy | **Methods, 3^rd^ paragraph:** Cohort I: “Controls deceased before the index date were excluded, and replaced when a new matching control who was alive on the index date could be found within three attempts. All individuals with index dates before January 1, 1988 were excluded from analyses, to provide a minimum retrospective study time of 1 year, and to allow 1 year to capture diagnoses of PD in individuals with disease onset before data collection began.”  **Methods, 4^th^ paragraph:** Cohort II: “pairs containing a control diagnosed with PD before the index date were excluded. Controls deceased before the index date were replaced when possible. Fallers for whom no matching control, alive at index date, could be found within 3 attempts were excluded. To allow for 1 year to capture diagnoses of PD, individuals with index date before January 1, 1988 were excluded from analysis.” |
|  |  | (*e*) Describe any sensitivity analyses | **Methods, 8^th^ paragraph:** “To assess potential confounding by the competing risk of death, we also performed the same analyses separately in a sub cohort (cohort IIb) where all matched pairs in which any individual died during follow up were excluded. All models were adjusted for sex and year of birth, education level, and comorbidities diagnosed before the index date.” |
| **Results** | | |  |
| Participants | 13* | (a) Report numbers of participants at each stage of study—eg numbers potentially eligible, examined for eligibility, confirmed eligible, included in the study, completing follow-up, and analysed | **Figure 1** |
|  |  | (b) Give reasons for non-participation at each stage | **Figure 1 and Methods section (paragraph 4 and 5)** |
|  |  | (c) Consider use of a flow diagram | **Figure 1** |
| Descriptive data | 14* | (a) Give characteristics of study participants (eg demographic, clinical, social) and information on exposures and potential confounders | **Table 1** |
|  |  | (b) Indicate number of participants with missing data for each variable of interest | **Methods, 5^th^ paragraph:** “…this information was available for 98.8% of individuals in cohort I and 97.8% of individuals in cohort II.”  **Table 1.** |
|  |  | (c) *Cohort study*—Summarise follow-up time (eg, average and total amount) | **Table 1**  **Results**, **1^st^ paragraph:** Cohort I: “median study period of 19.9 (range 1.0–25.0) years before the index date”  **Results**, **2^nd^ paragraph:** Cohort II: “median 5.8 (range 0–25.0) years” |
| Outcome data | 15* | *Cohort study*—Report numbers of outcome events or summary measures over time | **Table 2 and Table 3** |
|  |  | *Case-control study—*Report numbers in each exposure category, or summary measures of exposure | **Table 1**  **Results**, **1^st^ paragraph:** “Cohort I comprised 24,412 individuals with PD and 243,363 controls”  **Results**, **2^nd^ paragraph:** “Cohort II consisted of 622,333 fallers and 622,333 non-fallers” |
|  |  | *Cross-sectional study—*Report numbers of outcome events or summary measures |  |
| Main results | 16 | (*a*) Give unadjusted estimates and, if applicable, confounder-adjusted estimates and their precision (eg, 95% confidence interval). Make clear which confounders were adjusted for and why they were included | **Table 2 and Table 3**  **Legend to Table 2:** “Odds ratio for injurious falls (a) and for hip fractures (b) in cohort I, for individuals with Parkinson’s disease compared to controls, investigated by conditional logistic regression model adjusted for education level and comorbid diagnoses (dementia; stroke; myocardial infarction; diabetes mellitus; depression; alcohol dependency or abuse; drug dependency or abuse). The grey areas represent the 95% confidence intervals.”  **Legend to Table 3:** “Odds ratio (OR) for Parkinson’s disease (PD) after injurious fall in cohort II, investigated by conditional logistic regression model adjusted for education level and comorbid diagnoses (dementia; stroke; myocardial infarction; diabetes mellitus; depression; alcohol dependency or abuse; drug dependency or abuse). CI = confidence interval.”  **Methods, 5^th^ paragraph:** “Other diagnoses that might confound the associations studied were selected based on previous known associations with falls” |
|  |  | (*b*) Report category boundaries when continuous variables were categorized | **Methods, 5^th^ paragraph:** “As a proxy for socioeconomic situation, information about individuals’ education level (low, vocational school or ≤9 years primary school; high, university or ≥3 years secondary school)” |
|  |  | (*c*) If relevant, consider translating estimates of relative risk into absolute risk for a meaningful time period | **Table 2 and Table 3.** |
| Other analyses | 17 | Report other analyses done—eg analyses of subgroups and interactions, and sensitivity analyses | **Results**, **last paragraph:** “However, in the sub cohort excluding all pairs censored by death (S1 Table), no inverse relationship between falling and PD was observed (S2 Table, S1 Fig).” |
| **Discussion** | | |  |
| Key results | 18 | Summarise key results with reference to study objectives | **Discussion, 1^st^ paragraph:** “In general, we found that the risk of injurious falls was elevated a decade before the diagnosis of PD, and the risk of hip fracture was increased >15 years before this diagnosis. The strength of these associations between injurious falls, hip fracture, and PD increased distinctly up to the diagnosis of PD. The time-dependent patterns indicate a direct link between injurious falls and subsequent PD, and may be explained by subtle neurodegenerative impairment many years before the diagnosis of this disease.” |
| Limitations | 19 | Discuss limitations of the study, taking into account sources of potential bias or imprecision. Discuss both direction and magnitude of any potential bias | **Discussion, 4^th^ paragraph:** “First, data were obtained from registers and diagnoses could not be clinically confirmed for the present study, although all diagnoses were recorded in the context of specialist healthcare. It is likely that individuals with PD would more often be diagnosed in primary care in rural parts of Sweden, and therefore not be included as cases of PD in the present study. A similar effect could be expected for fall related injuries; less severe injuries that do not demand surgery are less likely be diagnosed in specialist care. Thus, the accuracy for hip fractures can be expected to be higher than for fall related injuries in general[24]. However, any incorrect or missed diagnosis would contribute to regression dilution bias, causing wider standard errors and attenuation of the associations found between PD and injurious falls toward zero[25]. Second, this observational study did not permit exploration of the mechanisms underlying the observed associations or documentation of a causal link between injurious falls and PD, although the time-dependent associations found strongly suggest such a relationship. Third, we lacked information about smoking status. This would have been of interest since smoking is associated with a reduced risk for PD[26], and a higher education is known to be associated with less smoking[27, 28]. Smoking status may therefore explain the association between higher education and PD in the present study.” |
| Interpretation | 20 | Give a cautious overall interpretation of results considering objectives, limitations, multiplicity of analyses, results from similar studies, and other relevant evidence | **Introduction, 2^nd^ paragraph:** “PD has an insidious onset; the cardinal motor symptoms are preceded by substantial neurodegeneration[6, 7], the timing of which remains uncertain[8, 9]. Several studies have reported increased occurrence of non-specific symptoms years or decades before the diagnosis of PD[8, 9]. These prodromal signs reported do typically not involve motor function, and balance impairment has been considered to be a late-stage symptom in PD”  **Discussion, last paragraph:** “In conclusion, we found that the risk of injurious falls in general, and especially those resulting in hip fracture, was increased decades before the diagnosis of PD. These findings suggest that clinically relevant neurodegenerative impairment could be present many years before the clinical onset of the disease. It would be of value if our results identifying markers of PD more than a decade before diagnosis, from the present and previous studies[16, 29], could be confirmed in other settings and in other countries. |
| Generalisability | 21 | Discuss the generalisability (external validity) of the study results | **Discussion, 4^th^ paragraph:** “as the individuals were drawn from a nationwide cohort, the external validity of the results is likely high.” |
| **Other information** | | |  |
| Funding | 22 | Give the source of funding and the role of the funders for the present study and, if applicable, for the original study on which the present article is based | **Metadata:** “No funding was received for this work.” |

*Give information separately for cases and controls in case-control studies and, if applicable, for exposed and unexposed groups in cohort and cross-sectional studies.

**Note:** An Explanation and Elaboration article discusses each checklist item and gives methodological background and published examples of transparent reporting. The STROBE checklist is best used in conjunction with this article (freely available on the Web sites of PLoS Medicine at http://www.plosmedicine.org/, Annals of Internal Medicine at http://www.annals.org/, and Epidemiology at http://www.epidem.com/). Information on the STROBE Initiative is available at www.strobe-statement.org.
