# Supplementary material for: Risk of Injurious Fall and Hip Fracture up to 26 y before the Diagnosis of Parkinson Disease: Nested Case–Control Studies in a Nationwide Cohort
Source: PLoS Med. 2016 Feb 2;13(2):e1001954. doi: 10.1371/journal.pmed.1001954 (PMC4737490; doi:10.1371/journal.pmed.1001954)
Supplement: S1 Table — (DOCX) [file pmed.1001954.s003.docx]

**S1 table.** **Descriptive data of cohort II after excluding all matched pairs censored by death during the study time (cohort IIb).**

| Cohort IIb | | | |
| --- | --- | --- | --- |
|  | **Fallers** | **Controls** | ***P*** |
| N | 383,169 | 383,169 |  |
| Study time [years; median (range)] | 5.8 (0–25.0) | 5.8 (0–25.0) | 0.669 |
| Age at index [years; median (range)] | 64.3 (32.1‒106.5) | 64.3 (32.1‒106.6) | 0.786 |
| Women (%) | 253,420 (66.1%) | 253,420 (66.1%) | 1.000 |
| Education level |  |  | <0.001 |
| High | 122,223 (31.9%) | 123,039 (32.1%) |  |
| Low | 257,185 (67.1%) | 256,795 (67.0%) |  |
| Missing | 3,761 (1.0%) | 3,335 (0.9%) |  |
| Comorbitity |  |  |  |
| Myocardial infarction | 15,333 (4.0%) | 13,694 (3.6%) | <0.001 |
| Stroke | 17,552 (4.6%) | 10,650 (2.8%) | <0.001 |
| Diabetes | 24,520 (6.4%) | 17,041 (4.5%) | <0.001 |
| Dementia | 7,445 (1.9%) | 2,924 (0.8%) | <0.001 |
| Depression | 13,353 (3.5%) | 7,565 (2.0%) | <0.001 |
| Alcohol abuse | 14,773 (3.9%) | 4,171 (1.1%) | <0.001 |
| Drug abuse | 2,757 (0.7%) | 959 (0.3%) | <0.001 |
| Incident PD during follow–up | 1,463 (0.4%) | 963 (0.3%) | <0.001 |
| Any fracture | 180,277 (47.1%) |  |  |
| Hip | 34,387 (9.0%) |  |  |
| Wrist | 67,387 (17.6%) |  |  |
| Lower leg | 54,950 (14.3%) |  |  |
| Humerus | 26,140 (6.8%) |  |  |
| Head injury | 26,230 (6.9%) |  |  |
