# Supplementary material for: Risk of Injurious Fall and Hip Fracture up to 26 y before the Diagnosis of Parkinson Disease: Nested Case–Control Studies in a Nationwide Cohort
Source: PLoS Med. 2016 Feb 2;13(2):e1001954. doi: 10.1371/journal.pmed.1001954 (PMC4737490; doi:10.1371/journal.pmed.1001954)
Supplement: S2 Table — OR estimated by conditional logistic regression model adjusted for education level and comorbid diagnoses (dementia, stroke, myocardial infarction, diabetes mellitus, depression, alcohol dependency or abuse, drug dependency or abuse). (DOCX) [file pmed.1001954.s004.docx]

### **S2 Table. Odds ratio for Parkinson’s disease according to fall, after excluding all matched pairs censored by death during the study time in cohort II (cohort IIb).**

| **Time interval** | **Participants at risk at**  **beginning of interval** | | **PD within interval** | | ***p*** | **Adjusted OR for PD**  **(95% CI),**  **according to fall** | ***P*** |
| --- | --- | --- | --- | --- | --- | --- | --- |
|  | **Fallers** | **Non-fallers** | **Fallers** | **Non-fallers** |  |  |  |
| **≥15 years after index** | 44,798 | 44,811 | 92 (0.21%) | 108 (0.24%) | *0.258* | 0.83 (0.62–1.12) | *0.222* |
| **10–15 years after index** | 94,488 | 94,545 | 111 (0.12%) | 109 (0.12%) | *0.889* | 0.96 (0.72–1.26) | *0.751* |
| **7–10 years after index** | 160,170 | 160,274 | 179 (0.11%) | 126 (0.08%) | *0.002* | 1.52 (1.19–1.93) | *0.001* |
| **5–7 years after index** | 210,031 | 210,178 | 166 (0.08%) | 116 (0.06%) | *0.003* | 1.42 (1.11–1.83) | *0.006* |
| **4–5 years after index** | 238,055 | 238,251 | 110 (0.05%) | 67 (0.03%) | *0.001* | 1.73 (1.25–2.38) | *0.001* |
| **3–4 years after index** | 269,624 | 269,780 | 128 (0.05%) | 89 (0.03%) | *0.008* | 1.50 (1.11–2.02) | *0.008* |
| **2–3 years after index** | 304,214 | 304,406 | 135 (0.04%) | 100 (0.03%) | *0.022* | 1.33 (1.00–1.76) | *0.047* |
| **1–2 years after index** | 343,071 | 343,252 | 185 (0.05%) | 115 (0.03%) | *<0.001* | 1.50 (1.16–1.93) | *0.002* |
| **3–12 months after index** | 372,315 | 372,451 | 173 (0.05%) | 93 (0.02%) | *<0.001* | 1.89 (1.44–2.47) | *<0.001* |
| **<3 months after index** | 383,169 | 383,169 | 184 (0.05%) | 40 (0.01%) | *<0.001* | 4.85 (3.31–7.10) | *<0.001* |

OR estimated by conditional logistic regression model adjusted for education level and comorbid diagnoses (dementia, stroke, myocardial infarction, diabetes mellitus, depression, alcohol dependency or abuse, drug dependency or abuse).
