# Supplementary material for: Risk of Injurious Fall and Hip Fracture up to 26 y before the Diagnosis of Parkinson Disease: Nested Case–Control Studies in a Nationwide Cohort
Source: PLoS Med. 2016 Feb 2;13(2):e1001954. doi: 10.1371/journal.pmed.1001954 (PMC4737490; doi:10.1371/journal.pmed.1001954)
Supplement: S1 Text — (DOCX) [file pmed.1001954.s005.docx]

**History of analysis plan**

The idea of the present study was based on the results of our previous study, where we found a reduced muscle strength in young adulthood in men who later developed PD[1]. The plan of the present study was designed in February 2015, when the analyses were conceived by Peter Nordström and Helena Nyström. The current version of the paper is in one way different to the original manuscript: on request from a reviewer (October 2015), we have removed one sensitivity analysis. That analysis investigated the risk of falls after the diagnosis of PD, and revealed that the relative risk of falls was most increased in the time closest to diagnosis of PD, with a subsequent gradual decline from 3 months after the diagnosis. Except for this exclusion, the current version of the paper do not deviate from the original study plan.

The original study plan included the setup of the 2 different nested case-control cohorts, and the definition of the time intervals used in the retrospective analyses in cohort I. We decided to keep the time intervals short in close relation to index to optimize the precision. Given the declining number of participants due to censoring at time points further form index, we allowed a gradually increasing length of the time intervals to retain statistical power. The length of intervals were adopted from our previous study concerning depression and subsequent risk of PD[2]. The original analysis plan also included a decision to adjust for comorbid disorders and education, which we did not change.

For the prospective analyses in cohort II, we initially tested the proportional hazards assumption for the association between fall and subsequent PD, and found that this assumption was not fulfilled. Therefore, further analyses were conducted with methods allowing the strength of association to vary with time and, for consistency, we chose the same time intervals as in the retrospective analyses of cohort I.

1. Gustafsson H, Aasly J, Stråhle S, Nordström A, Nordström P. Low muscle strength in late adolescence and Parkinson disease later in life. Neurology. 2015. doi: 10.1212/WNL.0000000000001534. PubMed PMID: 25841033.

2. Gustafsson H, Nordström A, Nordström P. Depression and subsequent risk of Parkinson disease: A nationwide cohort study. Neurology. 2015. doi: 10.1212/WNL.0000000000001684. PubMed PMID: 25995056.
